# A comparison of mitochondrial genomes from five species in three genera suggests polyphyly in the subfamily Achatinellinae (Gastropoda: Pulmonata: Stylommatophora: Achatinellidae)

**DOI:** 10.1080/23802359.2018.1473737

**Published:** 2018-05-18

**Authors:** Melissa R. Price, Zac H. Forsman, Ingrid Knapp, Robert J. Toonen, Michael G. Hadfield

**Affiliations:** a Department of Natural Resources and Environmental Management, University of Hawai‘i at Mānoa, Honolulu, HI, USA;; b Kewalo Marine Laboratory, Pacific Biosciences Research Center, University of Hawai‘i at Mānoa, Honolulu, HI, USA;; c Hawai‘i Institute of Marine Biology, University of Hawai‘i at Mānoa, Kāne‘ohe, HI, USA

**Keywords:** Endangered species, Hawaiian tree snail, mitogenome, Mollusca, RADseq

## Abstract

We compare the complete mitochondrial genomes of *Achatinella fulgens*, *A. mustelina, A. sowerbyana*, *Partulina redfieldi*, and *Perdicella helena*, five species of Hawaiian tree snails across three genera. Mitogenomes ranged in length from 15,187 to 16,793 base pairs, with a base composition of A (36.4–37.4%); T (42.2–42.7%); C (8.8–9.2%); and G (11.3–11.8%). Similar with other pulmonates, these mitogenomes contain 13 protein-coding genes, two ribosomal RNA genes, and 22 transfer RNA genes, with the order conserved among genera. Our study suggests polyphyly in the current arrangement of the subfamily Achatinellinae, part of a spectacular radiation in the Hawaiian Islands.

Hawaiian tree snails (Achatinellidae) are part of a spectacular radiation across six Hawaiian Islands (Pilsbry and Cooke 1912–1914). Habitat loss, predation by introduced species, and intensive harvesting by collectors led to the extinction of at least 75% of the species and resulted in the declaration of all remaining species in the genus *Achatinella*, as well as several in the genera *Partulina* and *Newcombia*, as Endangered, (Hadfield and Mountain 1980; U.S. Fish and Wildlife Service 1981; Hadfield 1986; U.S. Fish and Wildlife Service 2016).

We sequenced the complete mitochondrial genomes of *A. fulgens*, *Partulina redfieldi*, and *Perdicella helena* (GenBank accession numbers MG925058, MG925057, MH018243) for comparison with two previously published mitogenomes in the genus *Achatinella*. Small tissue samples were collected from two to three populations of each species (15–40 individuals per population), using non-lethal methods, and preserved in 100% ethanol until DNA extraction (Thacker and Hadfield 2000). DNA was individually extracted from tissue samples using a DNeasy Blood and Tissue Kit (Qiagen, Hilden, Germany) according to the manufacturer’s protocol. Extracted DNA was quantified using the Biotium AccuClear Ultra High Sensitivity dsDNA quantitation kit (Fresno, CA) with 7 standards.Individuals from within a population were pooled equimolarly and then, from these pools, libraries were prepared for genome scanning using the ezRAD protocol version 2.0 (Toonen et al. 2013; Knapp et al. 2016). The libraries were digested with the frequent cutter restriction enzyme DpnII from New England Biolabs^®^ (Ipswich, MA) and prepared for sequencing on the Illumina^®^ MiSeq (San Diego, CA) using the Kapa Biosystems Hyper Prep kit (Indianapolis, IN). All samples were amplified to generate 1 µg of adapter-ligated DNA, then validated and quantified to ensure equal pooling on the MiSeq flow cell, using a Bioanalyzer and qPCR. Quality control checks and sequencing were performed by the Hawaii Institute of Marine Biology Genetics Core Facility.

We obtained a range of 2,740,474–5,737,666 sequences per population. Reads were paired (PEAR; Zhang et al. 2013), then mapped to the mitogenome of *A. mustelina* (Price et al. 2016a) using Geneious 6.0 (Newark, NJ). In total, 4208–7171 reads, or ∼0.1% of reads per population, mapped to the mitochondrial genome, with coverage ranging from 1× to 1678× per site (mean coverage 99 ± 172). Annotation of mitochondrial elements was carried out with DOGMA (Wyman et al. 2004) and MITOS (Bernt et al. 2013).

The mitogenomes of species within Achatinellinae are similar with those of other pulmonates (White et al. 2011), with 13 protein-coding genes, two rRNA genes, and 22 tRNA genes. The mitogenome sizes are 15,346 (*A. fulgens*), 16,793 (*P. redfieldi*), and 15,187 bp (*P. helena*), in length, within 1200 bp of *A. mustelina* (Price et al. 2016b). Base compositions for each nucleotide ranged as follows: A (36.4–37.4%); T (42.2–42.7%); C (8.8–9.2%); and G (11.3–11.8%). *Achatinella mustelina* and *A. sowerbyana* grouped with *Perdicella helena*, whereas *A. fulgens* grouped with *Partulina redfieldi*, suggesting polyphyly in the current taxonomic arrangement of genera within Achatinellinae (Figure 1).

**Figure 1.**
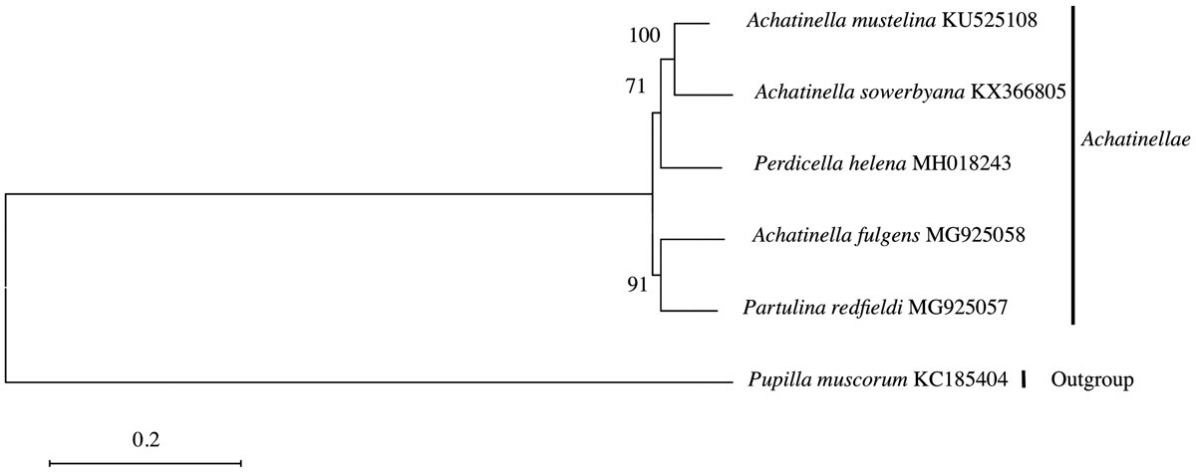
Placement of *Achatinella* with the Pupillidae out-group. Alignments, model tests, and maximum-likelihood analyses were performed using MEGA version 6.06 (Tamura et al. 2013). The mitochondrial genomes were aligned using Muscle in MEGA version 6.06 (Tamura et al. 2013). Default settings were used with the following exceptions: the refining alignment preset was run after initial default alignment. The nucleotide substitution model was found to be GTR + G + I using the Akaike Information Criterion (AIC). Maximum-likelihood analysis of the nucleotides was run using the identified model, with bootstrap support values based on 1000 replicates. The resulting tree suggests polyphyly in the current arrangement of the subfamily Achatinellinae (Price et al. 2016a, 2016b; ).

## Collection site

Samples were collected from 21.53742, –157.92099 (decimal degrees).
